# Species-specific Primer and Probe Sets for Detection of Syntrophic Long-chain Fatty Acid-degrading Bacteria in Anaerobic Digestion Using Quantitative PCR

**DOI:** 10.1264/jsme2.ME23023

**Published:** 2023-06-24

**Authors:** Riku Sakurai, Yasuhiro Fukuda, Chika Tada

**Affiliations:** 1 Laboratory of Sustainable Animal Environment, Graduate School of Agricultural Science, Tohoku University, Osaki, Miyagi, Japan

**Keywords:** quantitative PCR, long-chain fatty acid, *Syntrophomonas zehnderi*, *Syntrophomonas palmitatica*, anaerobic digestion

## Abstract

Lipid-rich wastes are energy-dense substrates for anaerobic digestion. However, long-chain fatty acids (LCFAs), key intermediates in lipid degradation, inhibit methanogenic activity. In this study, TaqMan-based qPCR assays targeting the 16S rRNA gene of the cardinal LCFA-degrading bacterial species *Syntrophomonas palmitatica* and *S. zehnderi* were developed and validated. A trial experiment showed the advantage of species-specific quantification versus genus-specific quantification in assessing bacterial capacity for lipidic waste degradation. These qPCR assays will serve as monitoring tools for estimating the LCFA-degrading capacity of anaerobic digester communities and developing an effective strategy to enrich LCFA-degrading bacteria.

Anaerobic digestion is a practical method for recovering energy resources, such as methane, by treating various organic wastes. However, the broader application of anaerobic digestion requires further improvements in treatment efficiency and stability (Salama *et al.*, 2019). The co-digestion of energy-rich wastes with wastewater sludge is attracting increasing attention due to improved digestion efficiency and biomethane yield (Wang *et al.*, 2013). Lipidic wastes are considered to be desirable co-substrates because they exhibit higher levels of convertibility to biogas than sugars and proteins (Xu *et al.*, 2018). However, long-chain fatty acids (LCFAs, with carbon chains of more than 12 carbon atoms) produced by the hydrolysis of lipids have been reported to inhibit anaerobic microorganisms (Shin *et al.*, 2003; Palatsi *et al.*, 2009; Silva *et al.*, 2016). Therefore, LCFA degradation is required to promote stable and effective methane production from lipidic wastes.

In an anaerobic environment, LCFAs are degraded to hydrogen and acetate via the beta-oxidation pathway (Elsamadony *et al.*, 2021). This process is the rate-limiting step during the anaerobic digestion of lipid-rich substrates (Lalman and Bagley, 2002). The syntrophic relationship between beta-oxidizing bacteria and methanogenic archaea is necessary because the reaction only proceeds under very low H_2_ pressure (Weng and Jeris, 1976). For example, Schink and Friedrich (1994) reported that the oxidation of 3-hydroxy butyryl CoA to acetoacetyl CoA may be coupled to proton reduction at a hydrogen partial pressure close to 10^–5^ bar [=1 Pa].

Currently, all of the isolated bacterial species that grow on LCFAs with methanogens belong to the families Syntrophomonadaceae and Syntrophaceae (Elsamadony *et al.*, 2021). The bacterial species detected in mesophilic anaerobic digestion are *Syntrophomonas sapovorans* (Roy *et al.*, 1986), *S. wolfei* subsp. *saponavida* (Lorowitz *et al.*, 1989), *S. curvata* (Zhang *et al.*, 2004), *S. zehnderi* (Sousa *et al.*, 2007), *S. palmitatica* (Hatamoto *et al.*, 2007a), and *Syntrophus aciditrophicus* (Jackson *et al.*, 1999). Previous studies indicated that, among these species, *Syntrophomonas palmitatica* and *S. zehnderi* are the most important for LCFA degradation. Hatamoto *et al.* (2007b) investigated LCFA-degrading microorganisms using RNA-based stable isotope probing with ^13^C-labeled palmitate (saturated LCFA) and demonstrated that *S. palmitatica* was the dominant bacterial species. We also showed that after an approximately two-year pre-incubation of anaerobic digester sludge, the dominant species of genus *Syntrophomonas* was the closest to *S. palmitatica*, and its abundance increased as the loading amount of an oily substrate became higher (Sakurai *et al.*, 2021). Similarly, a previous study indicated that *S. zehnderi*-related species became dominant in a continuously fed anaerobic digester treating LCFA (Ziels *et al.*, 2017); therefore, *S. palmitatica* and *S. zehnderi* both dominate the degradation of LCFAs. However, methods to quantify these species have been limited to full-length 16S rRNA amplicon sequencing, shotgun metagenomics, and metatranscriptomics, all of which are expensive and time-consuming.

Quantitative PCR (qPCR) is a widely-employed, highly sensitive method for rapidly quantifying microorganisms of interest (Nadkarni *et al.*, 2002). It is a proven and powerful tool for monitoring microbial populations in anaerobic digesters (Yu *et al.*, 2005; Ziels *et al.*, 2015). The use of a dual-labeled fluorogenic hydrolysis probe in the qPCR assay (TaqMan based) offers more specific detection than the SYBR Green assay (Wittwer *et al.*, 1997). A previous study developed a TaqMan-based qPCR assay targeting the genus *Syntrophomonas* (Ziels *et al.*, 2015). However, since not all *Syntrophomonas* members are capable of degrading LCFAs, species-specific quantification assays are needed to evaluate the LCFA-degrading potential of anaerobic digester communities. The present study aimed to develop and validate TaqMan qPCR assays targeting the two most important LCFA-degrader microorganisms during anaerobic digestion: *S. palmitatica* and *S. zehnderi*.

The developed primer/probe sets targeting the *S. palmitatica* and *S. zehnderi* 16S rRNA genes are listed in Table 1, and the amplicon sizes for their qPCR assays were 256 and 420 bp, respectively. The specificity of each primer and probe set was tested *in silico* using SILVA TestPrime 1.0 and TestProbe 3.0 (Klindworth *et al.*, 2013). Microbial organisms with three or more mismatches between each of the three nucleotide sequences were not identified and this was attributed to the low possibility of their amplification (Yu *et al.*, 2005). As a result, no potential non-target organisms were detected by the *in silico* ana­lysis for the *S. palmitatica* or *S. zehnderi* qPCR assays. To further verify the specificity of the primer/probe set, experimental validation was conducted as follows. PCR was performed using the primer sets developed for *S. palmitatica* and *S. zehnderi*. PCR products were purified and cloned into T-Vector pMD19 (Simple) (TaKaRa Bio), and the resulting vectors were transformed into T-Competent Quick DH5a (TOYOBO). Positive transformants were randomly selected and colony PCR was performed using the M13 primer RV (5′-CAGGAAACAGCTATGAC-3′) and M13 primer M4 (5′-GTTTTCCCAGTCACGAC-3′) (TaKaRa Bio). Clones with inserted DNA were identified and successfully sequenced after purification using ExoSAP-IT (Thermo Fisher Scientific). The insertion sequences were aligned using MAFFT v7.475 (Katoh and Standley, 2013), and primer sequences were trimmed using Jalview v 2.9.0b2 (Waterhouse *et al.*, 2009). The taxa from which the sequences arose were identified using BLAST (http://www.blast.ncbi.nlm.nih.gov/genbank/). Using these clone sequences as templates, TaqMan-based qPCR assays were conducted to confirm that the assays successfully detected the target sequences only. The 16S rRNA nucleotide sequences obtained in the present study were deposited in Genbank/EMBL/DDBJ under accession codes LC752552 to LC752640.

Using the primer sets for the* S. palmitatica* qPCR assay, 37 clones were constructed and sequenced (Table S1): 18 clones for *S. palmitatica* (identity: 92–100%) and 19 for *S. zehnderi* (identity: 91–100%). There were 16 clones with the TaqMan probe sequence with fewer than three mismatches from the clone library. These clones were the closest to *S. palmitatica* (identity: 99–100%). Only these clones were amplified by qPCR targeting *S. palmitatica* (Fig. 1A). Using the primer sets for the *S. zehnderi* qPCR assay, 52 clones were constructed and sequenced (Table S2): 18 clones for *S. zehnderi* (identity: 93–99%), four for *S. bryantii* (identity: 95%), 19 for *S. sapovorans* (identity: 95–99%), and 11 for *S. wolfei* (identity: 93–100%) and 4 for *S. bryantii* (identity: 95%). Five clones carrying the region matching the TaqMan probe had less than three mismatches from the clone library. The species closest to these clones was *S. zehnderi* (identity: 97–99%). Only these clones were amplified by qPCR targeting *S. zehnderi* (Fig. 1B). Overall, the primer/probe sets developed for *S. palmitatica* or *S. zehnderi* successfully amplified only the target sequences. These results indicate the high specificity of the qPCR assays developed. DNA samples for calibration standards were prepared by cloning the target PCR amplicon fragment of each primer set, derived from pure culture DNA, using the same protocol described above. Pure *S. palmitatica* cultures were purchased from the National Institute of Technology and Evaluation (Tokyo, Japan). Pure‍ ‍*S. zehnderi* cultures were purchased from Deutsche Sammlung von Mikroorganismen und Zellkulturen GmbH (Braunschweig, Germany). The slopes of the calibration dilution series were calculated using linear regressions by plotting Ct values versus template concentrations. A linear dynamic range spanning seven orders of magnitude (10^8^–10^2^ copies) is displayed for each species (Fig. 2). Slopes of –‍3.016 for *S. palmitatica* and –3.024 for *S. zehnderi* were obtained. The qPCR calibration slopes for the *S. palmitatica* and *S. zehnderi* assays corresponded to PCR efficiencies of 114.6 and 114.1%, respectively. These values were within the acceptable 80–120% range suggested in the MIQE guidelines (Bustin *et al.*, 2009). The regression coefficients (R^2^) for *S. palmitatica* and *S. zehnderi* were 0.990 and 0.996, respectively.

The utility of the qPCR assays developed was assessed using the two bioreactor samples described in our previous study: Sludge I and Sludge II (Sakurai *et al.*, 2021). Briefly, Sludge I was prepared by incubating the collected sludge with lipidic waste for approximately 2 years. Sludge II was prepared by incubating it with glucose. In a batch experiment using scum from digested lipidic waste, Sludge I showed a 30% higher methane conversion rate than Sludge II. These sludges were used in the qPCR assay for specific 16S rRNA genes of the genus *Syntrophomonas* and species *S. palmitatica* and *S. zehnderi* (Table 2). Using the genus-specific primer/probe set developed by Ziels *et al.* (2015), the 16S rRNA gene copy numbers of *Syntrophomonas* were 3.9±0.9 E+05 in Sludge I and 2.2±0.2 E+06 copies mL^–1^ in‍ ‍Sludge II. The 16S rRNA gene copy number of *S. palmitatica* was 9.1±0.9 E+05 copies mL^–1^ in Sludge I and not detected in Sludge II. The 16S rRNA gene copy number of *S. zehnderi* was 2.9±0.3 E+05 copies mL^–1^ in Sludge I and not detected in Sludge II. Even though Sludge II showed a higher 16S rRNA gene copy number of *Syntrophomonas*, neither *S. palmitatica* nor *S. zehnderi* were detected. These results suggested that the presence of *S. palmitatica* and *S. zehnderi* in Sludge I contributed to a 30% higher methane conversion rate. In conclusion, the presented TaqMan-based qPCR assays targeting *S. palmitatica* and *S. zehnderi* were validated and applied. Field testing indicated the advantage of species-specific quantification towards genus-specific quantification when assessing the capability of lipidic waste degradation. The assays presented here enabled the absolute quantification of LCFA-degrading bacteria for the first time and may lead to the establishment of better anaerobic digester operating strategies.

## Citation

Sakurai, R., Fukuda, Y., and Tada, C. (2023) Species-specific Primer and Probe Sets for Detection of Syntrophic Long-chain Fatty Acid-degrading Bacteria in Anaerobic Digestion Using Quantitative PCR. *Microbes Environ ***38**: ME23023.

https://doi.org/10.1264/jsme2.ME23023

## Supplementary Material

Supplementary Material

## Figures and Tables

**Fig. 1.**
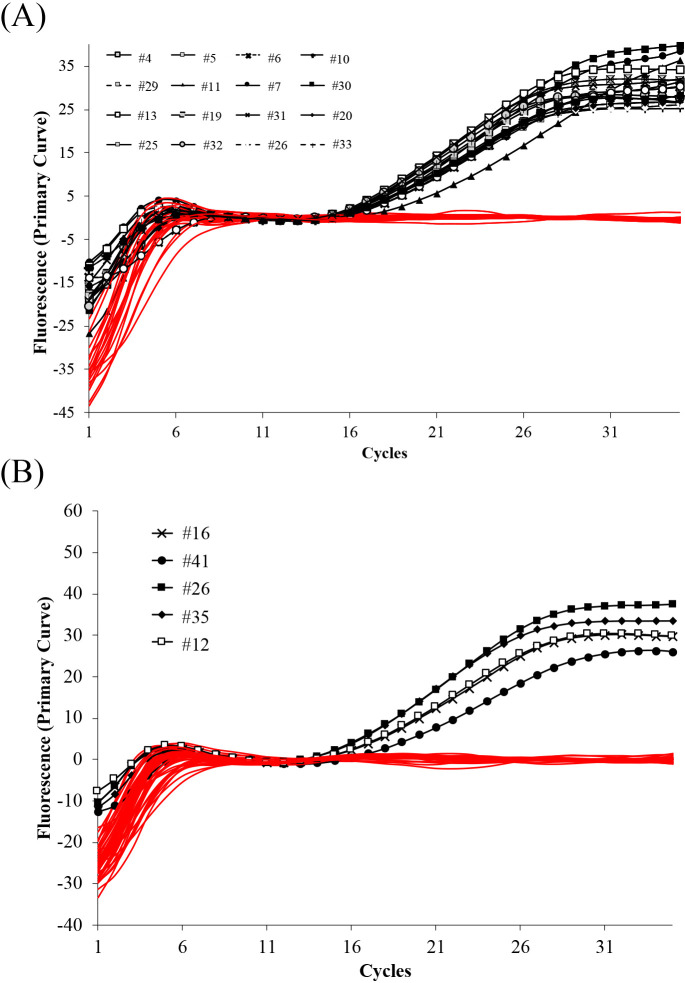
Specific detection of target species as increases in fluorescence using the clone library. TaqMan-based qPCR assays targeting (A) *Syntrophomonas palmitatica* and (B) *Syntrophomonas zehnderi*. Samples colored in red did not produce a fluorescence signal. The number following # indicates the clone ID described in Supplementary Table S1.

**Fig. 2.**
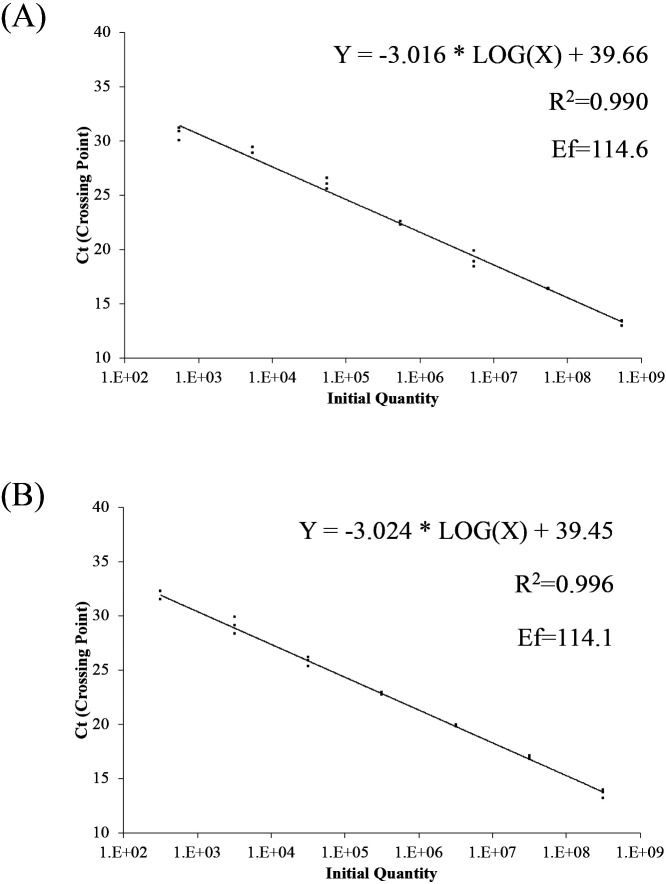
Threshold cycle (Ct) values versus 16S rRNA gene copies on a typical standard calibration curve. Standard curves generated from qPCR assays targeting (A) *Syntrophomonas palmitatica* and (B) *Syntrophomonas zehnderi*. Triplicate points are shown for each standard dilution.

**Table 1.** Characteristics of 16S rRNA gene-targeted qPCR primer/probe sets

	Target	Oligo. sequence (5′-3′)	*E. coli* position	Product size (bp)	Tm (°C)^b^
SynPal_Probe (P)	*Syntrophomonas palmitatica*	TGTCTAGAGCAATAACACGGCTTTAGAA	547–574		67
SynPalF (F)		GCGAAGAAGGCCTTAGGGTT	502–521	256	60
SynPalR (R)		CCTGCCCTCAAGAACTCCAG	738–757		60
SynZehn_Probe (P)	*Syntrophomonas zehnderi*	TGTTCGCGTCAGTAATATGGGCGTGAAA	509–542		75
SynZehnF (F)		GATGAGCCCGCGTCTGATTA	269–288	420	60
SynZehnR (R)		CAGTTTCAAGTGCAACCCCG	669–688		60

^a^ Designations in parentheses: P, TaqMan probe; F, forward primer; R, reverse primer. ^b^ Calculated using the nearest-neighbor method.

**Table 2.** Quantification of LCFA-degrading bacteria in anaerobic digestion sludge using qPCR assays developed in this study

Sample source	Target	Number of the 16S rRNA gene (copies mL^–1^*)	Reference
Sludge I	*Syntrophomonas*	3.9±0.9 E+05	Sakurai *et al.*, 2021
	*S. palmitatica*	9.1±0.9 E+05	This study
	*S. zehnderi*	2.9±0.3 E+05	This study
Sludge II	*Syntrophomonas*	2.2±0.2 E+06	Sakurai *et al.*, 2021
	*S. palmitatica*	n.d.	This study
	*S. zehnderi*	n.d.	This study

* Values represent the standard deviation of the mean (*n*=3 biological replicates).
